# Association of Birth Weight Centiles and Gestational Age With Cognitive Performance at Age 5 Years

**DOI:** 10.1001/jamanetworkopen.2023.31815

**Published:** 2023-08-31

**Authors:** Robert Eves, Dieter Wolke, Juliane Spiegler, Sakari Lemola

**Affiliations:** 1 Fakultät für Psychologie und Sportwissenschaft, Universität Bielefeld, Bielefeld, Germany; 2 Department of Psychology, Lifespan Health and Wellbeing Group, University of Warwick, Warwick, England, United Kingdom; 3 Kinderklinik, Universitätsklinikum Würzburg, Würzburg, Deutschland

## Abstract

**Importance:**

Birth weight percentiles (BWPs) are often dichotomized at the 10th percentile and show statistically significant association with later cognitive performance, for both preterm and term-born children. However, research testing nonlinear associations between BWPs and cognitive performance is scarce.

**Objective:**

To investigate culturally invariant, nonlinear associations of BWPs and gestational age with later cognitive performance.

**Design, Setting, and Participants:**

In this cohort study, participants with valid neonatal and cognitive data were combined from 4 observational cohorts, including the Millennium Cohort Study, the National Longitudinal Survey of Youth 1979 Child and Young Adult cohort, Growing Up in Ireland, and the Longitudinal Study of Australian Children, with children born between 2000 and 2002, 1980 and 2010, 2007 and 2008, and 2003 and 2004, respectively. Neonatal data were parent reported before age 1 year. At approximately 5 years of age, multiple cognitive tests were performed. Follow-up at 5 years of age was the predominant focus. Data were analyzed July 17, 2023.

**Exposure:**

The parent-reported neonatal data were used to calculate BWPs according to the Fenton growth chart.

**Main Outcome and Measure:**

Scores for IQ were created from multiple measures of cognition, which were *z* standardized separately within each cohort.

**Results:**

Of 30 643 participants (50.8% male), 7.5% were born preterm (before 37 weeks gestation) and 92.5% were term born (between 37 and 42 weeks gestation). In the pooled data using multivariate adaptive regression splines, IQ linearly increased by 4.2 points as BWPs increased from the first to the 69th percentile before completely plateauing. For gestational age, IQ linearly increased by 1.3 points per week up until 32 weeks, with the association reducing to 0.3 points per week after 32 weeks. The association of BWP with IQ was not moderated by gestational age. For term-born infants, the estimated IQ score was only clinically meaningfully lower than average when birth weight was below the third percentile. Consistent results were found when instead using multivariable regression where gestational age and BWPs were categorized into groups.

**Conclusions and Relevance:**

In this cohort study, lower BWPs and gestational age were independently associated with lower IQ. For term-born infants, a cutoff of the third percentile would be more appropriate than the traditionally used 10th percentile when the aim is estimating meaningful cognitive differences.

## Key Points

**Question:**

Are birth weight percentiles and gestational age nonlinearly associated with cognitive performance?

**Findings:**

In this cohort study of 4 longitudinal cohorts with 30 643 pooled participants, IQ was associated with increasing birth weight *z* scores until the *z* score equaled the 69th percentile, with a subsequent plateau. In contrast, every week of gestation was significantly associated with increased IQ, but the strongest association was observed in the very preterm range, before 32 weeks gestation.

**Meaning:**

Using the 10th birth weight percentile to estimate low cognitive performance does not adequately represent the nonlinear association between the 2 constructs.

## Teaser

This cohort study investigates culturally invariant, nonlinear associations of birth weight percentiles and gestational age with later cognitive performance.

## Introduction

Being born small for gestational age (SGA), often defined as a birth weight percentile (BWP) of lower than 10% for gestation, has been repeatedly shown to be associated with lower scores on intelligence tests (IQ)—an important predictor of a variety of long-term outcomes such as academic and occupational achievement, as well as physical health^1,2^—relative to those born appropriate for gestational age (AGA).^3,4^ The 10th percentile is often clinically used but may be arbitrary for associations with cognitive outcomes.^5,6^ If the 10th percentile is a valid cutoff for predicting IQ, then IQ scores may be substantially lower from the first to the 10th percentiles, with a subsequent step change and higher levels afterward. Alternatively, if one assumes a linear relationship between BWP and IQ, then for the 10th percentile to be a clinically useful, binary predictor of IQ, it should be associated with a standardized difference of at least 0.2 (3 IQ points).

The alternative is to consider continuous measures of relative birth weight such as BWPs or birth weight *z* scores (BWZS). Indeed, it has been shown that improved developmental outcomes are associated with increasing BWPs.^7,8^ However, analyses using continuous measures of relative birth weight that test for linear associations assume that there are no areas in the distribution where IQ scores substantially increase, plateau, or even decrease,^7,9^ assuming that the 100th BWP is optimal. As infants weighing above the 90th percentile have been found to have substantially higher risk of asphyxia and malignant neoplasms,^10^ increasingly higher BWPs may be associated with lower IQ, indicating a nonlinear association. Indeed, 1 study reported the optimal BWP for educational outcomes at the 66th percentile, with a subsequent decline in educational performance if BWP was higher.^8^ However there are contrasting findings, including no reduction in school performance from the 80th to the 100th BWP^11^ or that children with a BWP above 90% have higher IQs than those between the 50th and 90th percentiles.^12^

It has also been hypothesized that SGA/lower BWP’s negative association with IQ may be greater for infants born moderately/late preterm (MLP; between 32 and 37 weeks gestation) or very preterm (VP; <32 weeks gestation) relative to term born (between 37 and 42 weeks gestation).^13^ This is due to evidence that SGA status in preterm infants may more often result from intrauterine growth restriction, whereas SGA status in those born at term may more often result from being constitutionally small.^14^ However, a meta-analysis found that the relative IQ difference between SGA and appropriate for gestational age individuals to be of largely similar magnitude for those born preterm and at term.^3^ Unknown is whether this is equally true for those born VP specifically.

The aim of this cohort study was 2-fold. First, to test whether IQ increases as BWP increases and whether the association is nonlinear. Second, to determine whether the association between BWP and IQ differs by term-born, MLP, and VP status.

## Methods

### Sample and Procedure

Data from 4 large, longitudinal cohorts from high-income, English-speaking countries were pooled together. These included the Millennium Cohort Study (MCS) from the UK,^15,16^ the Longitudinal Study of Australian Children (LSAC) from the Commonwealth of Australia,^17,18^ the National Longitudinal Survey of Youth 1979 (NLSY79) Child and Young Adult cohort from the US,^19,20^ and the Growing Up in Ireland study (GUI) from the Republic of Ireland.^21,22^ Included participants from the MCS, LSAC, NLSY79 Child and Young Adult cohort, and GUI were born between 2000 and 2002, 2003 and 2004, 1980 and 2010, and 2007 and 2008, respectively. All studies were reviewed by institutional review boards or ethics committees, and written informed consent was collected from parents.^23,24,25,26^ All data are open access and, except for the NLSY79 Child and Young Adult cohort, thought to be nationally representative.^15,17,21,27^ This study followed the Strengthening the Reporting of Observational Studies in Epidemiology (STROBE) reporting guideline.

### Gestational Age and Birth Weight

In all cohorts, gestational age and birth weight information was collected from the primary caregiver at the first wave of data collection. In the MCS, this has been subsequently verified by matching the data to official birth records, finding them to be highly accurate, within 100 g in 92% of cases and within a week of gestation in 94% of cases.^28,29^ However, the GUI data are grouped together for infants with birth weights lower than 2500 g, while for all other participants it is to the nearest 100 g. Because BWPs could therefore not be accurately calculated for these participants with lower birth weights, they were excluded. Furthermore, in all cohorts, participants with a gestation equal to or greater than 42 weeks were removed.

### Birth Weight Percentile/*z* Score Determination

Using gestational age, birth weight, and the participants’ sex, the Fenton reference was used to determine BWZS.^30^ The Fenton reference uses data from 3 986 456 infants from multiple countries to develop an international growth chart that identifies the average birth weight at each gestational week. Participants with BWPs lower than 0.003% (*z* score <−4) or greater than 0.998% (*z* score >4) were removed, as these likely reflect reporting errors.^31^

### Cognitive Outcomes: Full-Scale IQ

To develop a full-scale IQ score, scores on standardized cognitive tests that broadly assessed crystallized and fluid intelligence were combined. Once scores for fluid and crystallized intelligence were calculated, the average of the 2 scores was taken as to calculate a full-scale IQ score, which was *z* score standardized separately in each cohort.

All cognitive tests were measured at approximately 5 years of age, with the exception of fluid intelligence measured at 7 years of age in the LSAC. To assess fluid intelligence in the MCS, the principal component of scores on the British Ability Scales (BAS) Picture Similarities and Pattern Construction tests was taken.^32,33^ In the LSAC, the Wechsler Intelligence Scale for Children, Fourth Edition’s Matrix Reasoning test was administered.^34,35^ In the NLSY79 Child and Young Adult cohort, the principal component of the Peabody Individual Achievement Test’s Mathematics and Reading Recognition was used.^36^ In the GUI, the BAS Picture Similarities test was used.^32^

To measure crystallized intelligence, the MCS and GUI used BAS’s Naming Vocabulary test.^32^ In the LSAC, the Peabody Picture Vocabulary Test, Third Edition was used,^37^ whereas in the NLSY79 Child and Young Adult cohort the revised Peabody Picture Vocabulary Test was used.^38^

### Confounders

Potentially confounding factors were based on those used in similar past studies and which were harmonizable across all cohorts.^8,12^ Confounders were child sex, English as the primary language for the child, maternal age, maternal height, maternal weight, maternal marital status, parity, maternal education, the household income, and cohort. All confounders were measured at the nearest assessment to birth, except for the child’s primary language, which was assessed at age 5 years.

### Statistical Analysis

All analyses were performed in R, version 4.1.1 (R Foundation). The primary analysis tested BWZS, gestational age centered on 40 weeks, cohort, their interaction terms, and the aforementioned confounders as factors associated with IQ *z* scores at 5 years of age. Birth weight *z* scores were used rather than BWPs because BWZS are naturally centered, which is advantageous for testing interactions.^39^ To test for nonlinear associations and to determine feature selection, multivariate adaptive regression splines (MARS) in the R package earth were used.^40^ As such, BWZS and gestational age were tested for “hinges,” where the association with IQ changes in strength and/or direction. For feature selection, MARS will only include features (eg, a linear effect of a confounder or an interaction term between BWZS, gestational age, or cohort), if they considerably improve the model fit, as determined by cross-validation. In cross-validation, 5 folds (subsets) of the data were created. Iteratively, 4 folds of the data were used to train models with a varying number of features, then tested on the fifth fold. The optimal model was determined by the highest mean *R*^2^ for the fifth fold (ie, the best average estimation on test data sets).

Rather than using MARS, a multivariable regression was performed as a secondary analysis with BWPs and gestational age categorized into traditional groups. The BWPs based on the Fenton *z* scores were categorized into 6 groups (<10%, ≤10% to <25%, ≤25% to <50%, ≤50% to <75%, ≤75% to <90%, and ≥90%). Gestational age was categorized into 3 groups: VP, MLP, and term born. The regression model included these categorical variables, their interaction terms, and the aforementioned confounders as factors associated with IQ *z* score at 5 years of age. From the regression results, estimated marginal means were calculated and contrasted as to test for the association of BWP with IQ within each gestation group, which was adjusted for multiple comparisons using the false discovery rate.^41^ The multivariable regression was 2-sided with *P* < .05 indicating statistical significance.

Finally, the coefficients of the MARS analysis were used to determine what combination of gestational age and BWZS would be associated with reduction of IQ scores by a clinically meaningful amount. Estimated IQ *z* scores of −0.2 (IQ, 97), −0.5 (IQ, 92.5), or −0.8 (IQ, 88) were thought to be equivalent to a small, medium, or large reduction in absolute IQ, respectively.^42^

Analyses only included participants with full data on IQ and BWZS, while missing confounder data were imputed by multiple chained equations.^43^ Three percent of covariate data was imputed, with 77% of participants having no missing covariate data. Five imputed data sets were used for the multivariable regression, while a single version of the imputed data was used in the MARS analysis.

## Results

### Participant Demographics

In the included sample, there were 30 643 participants with valid IQ scores at age 5 years and BWZS. Further perinatal and sociodemographic factors by cohort are summarized in Table 1. Of the 30 643 participants, 28 355, 2032, and 256 were term born, MLP, and VP, respectively (Table 1). When comparing cohorts, there were statistically significant but small differences regarding gestational age and BWZS (Table 1). Similarly, the BWZS and gestational age of the initial birth sample in each cohort were minimally lower relative to the final included sample (eTables 1 and 2 and eFigures 1-4 in Supplement 1).

**Table 1.** Perinatal and Sociodemographic Factors by Cohort

Characteristic	No. (%)				
	GUI (Ireland) (n = 7290)	LSAC (Australia) (n = 3738)	MCS (UK) (n = 14 072)	NLSY79 Child and Young Adult cohort (US) (n = 5543)	Total (N = 30 643)
Gestational age					
Term born	7062 (96.9)	3509 (93.9)	12 939 (91.9)	4845 (87.4)	28 355 (92.5)
Moderately/late preterm	228 (3.1)^a^	203 (5.4)	984 (7.0)	617 (11.1)	2032 (6.6)
Very preterm	0^a^	26 (0.7)	149 (1.1)	81 (1.5)	256 (0.8)
Gestational age, mean (SD), wk	39.5 (1.34)	39.1 (1.78)	39.3 (1.97)	38.5 (1.93)	39.1 (1.84)
Fenton birth weight percentile					
<10%	415 (5.7)	295 (7.9)	1729 (12.3)	548 (9.9)	2987 (9.7)
≤10%-<25%	1081 (14.8)	519 (13.9)	2497 (17.7)	727 (13.1)	4824 (15.7)
≤25%-<50%	1901 (26.1)	1049 (28.1)	3853 (27.4)	1342 (24.2)	8145 (26.6)
≤50%-<75%	2058 (28.2)	1042 (27.9)	3433 (24.4)	1454 (26.2)	7987 (26.1)
≤75%-<90%	1187 (16.3)	540 (14.4)	1690 (12.0)	865 (15.6)	4282 (14.0)
≥90%	648 (8.9)	293 (7.8)	870 (6.2)	607 (11.0)	2418 (7.9)
Fenton birth weight *z* score, mean (SD)	0.07 (0.89)	0.01 (0.93)	−0.18 (0.98)	−0.01 (1.08)	−0.06 (0.99)
Parity (nonfirstborn)	4549 (62.4)	2273 (60.8)	8134 (57.4)	3320 (59.9)	18 282 (59.7)
English as first language	6586 (90.3)	3400 (91.0)	12 857 (91.4)	4550 (82.1)^b^	27 393 (89.4)
Child sex					
Female	3611 (49.5)	1822 (48.7)	6908 (49.1)	2733 (49.3)	15 074 (49.2)
Male	3679 (50.5)	1916 (51.3)	7164 (50.9)	2810 (50.7)	15 569 (50.8)
Maternal height, mean (SD), cm	164 (6.72)	165 (7.31)	164 (7.09)	163 (6.99)	164 (7.03)
Maternal weight, mean (SD), kg	67.5 (12.6)	68.8 (15.0)	63.6 (12.7)	62.1 (13.7)	64.9 (13.3)
Maternal age, mean (SD), y	32.1 (5.16)	31.5 (5.07)	29.5 (5.88)	25.9 (5.10)	29.7 (5.87)
Mother is not university educated	5088 (69.8)	2387 (63.9)	11 642 (82.7)	3660 (66.0)	22 777 (74.3)
Family household income					
Low	2441 (33.5)	984 (26.3)	5092 (36.2)	1178 (21.3)	9695 (31.6)
Medium	2846 (39.0)	1597 (42.7)	4939 (35.1)	2674 (48.2)	12 056 (39.3)
High	2003 (27.5)	1157 (31.0)	4041 (28.7)	1691 (30.5)	8892 (29.0)
Full IQ, mean (SD), *z* scored	0 (1.00)	0 (1.00)	0 (1.00)	0 (1.00)	0 (1.00)

Abbreviations: GUI, Growing Up in Ireland; LSAC, Longitudinal Study of Australian Children; MCS, Millennium Cohort Study; NLSY79, National Longitudinal Survey of Youth 1979.

^a^ GUI participants with a birth weight less than 2500 g were removed, which subsequently increased the average week of gestation and reduced the number of those born moderately/late preterm and very preterm.

^b^ Missing data on the original NLSY79 variable were supplemented with information on whether the mother primarily spoke a language other than English during her own childhood.

### MARS Analysis: Nonlinear Associations Between BWZS, Gestational Age, and IQ

In adjusted models, there was a statistically significant association between BWZS and IQ scores, from a BWZS of −4 until a hinge point at *z* = 0.50 (69th BWP) (Table 2). For every 1 SD increase in BWZS, IQ *z* scores rose by 0.10. After this point, there was no association between BWZS and IQ, indicating a complete plateauing effect. Gestational age was also positively associated with IQ. From the lowest gestation of 25 weeks until 32 weeks, IQ *z* scores increased at 0.09 per week. Following this, there was a hinge point that indicated a plateauing effect with IQ *z* scores minimally increasing by 0.02 per week. Results indicated no evidence for statistically significant interactions between BWZS, gestational age, and cohort. Furthermore, results indicated that a number of social-economic factors were meaningfully associated with IQ (Table 2).

**Table 2.** Features Selected in the MARS Analysis as Meaningfully Associated With IQ *z* Score^a^

Feature	β Coefficient	GCV^b^
High income [Reference: low income]	0.39	100
Medium income [Reference: low income]	0.23	52
Primary language other than English [Reference: English]	−0.57	81
Maternal education: university educated [Reference: not university educated]	0.24	64
Parity: multiparous [Reference: primiparous]	−0.23	41
Child sex: male [Reference: female]	−0.13	35
BWZS until *z* = 0.50 (69th percentile)	0.10	30
Gestational age up to 32 wk	0.09	16
Gestational age after 32 wk	0.02	16
Maternal age (years)	0.009	24
Maternal weight (kg)	−0.003	11
Maternal marital status: not married [Reference: married]	−0.11	20

Abbreviations: BWZS, birth weight *z* score; GCV, generalized cross validation; MARS, multivariate adaptive regression splines.

^a^ Nonselected (ie, not meaningful) features include maternal height, cohort, and all interaction terms between cohort, BWZS, and gestational age.

^b^ GCV is a metric used in MARS analyses to indicate the relative importance of each factor included in the final model (eg, 100 = most important factor).

### Multivariable Regression: Association Between BWP Categories, Gestation Groups, and IQ

**Figure 1.**
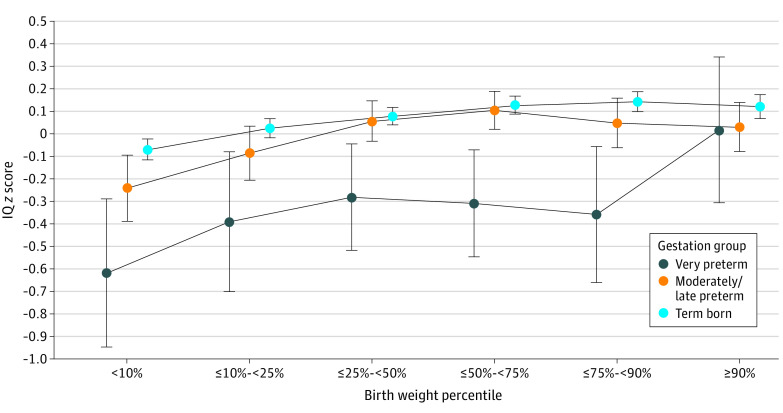
Estimated Marginal Means By Birth Weight Percentile and Gestation Group Error bars indicate 95% CIs.

Following the MARS analysis, the analysis was repeated using multivariable regression (eTable 3 in Supplement 1), from which the estimated marginal means were calculated (Figure 1) and contrasted (Table 3). For term borns, participants in the 75th to 90th BWP reference group had statistically significant higher IQ scores than those in the lowest 10%, the 10th to 25th percentiles, and the 25th to 50th percentiles. Among those born MLP, participants with BWPs in the 75th to 90th percentile reference group had statistically significant higher IQ scores than those in the lowest 10%. Among those born VP, the IQ *z* scores of the 75th to 90th BWP reference group were not statistically significantly different from any other BWP group.

**Table 3.** Contrast Analysis of Birth Weight Percentile Groups Within Each Gestational Age Group

Gestation group	Birth weight percentile comparison	Estimated IQ *z* score difference (SE)	Adjusted *P* value
Term born	<10% vs ≤75%-<90%	−0.21 (0.02)	<.001
Term born	≤10%-<25% vs ≤75%-<90%	−0.12(0.02)	<.001
Term born	≤25%-<50% vs ≤75%-<90%	−0.06 (0.02)	.002
Term born	≤50%-<75% vs ≤75%-<90%	−0.02 (0.02)	.61
Term born	≥90% vs ≤75%-<90%	−0.02 (0.03)	.61
Very preterm	<10% vs ≤75%-<90%	−0.26 (0.23)	.54
Very preterm	≤10%-<25% vs ≤75%-<90%	−0.03 (0.22)	.91
Very preterm	≤25%-<50% vs ≤75%-<90%	0.08 (0.19)	.91
Very preterm	≤50%-<75% vs ≤75%-<90%	0.05 (0.19)	.91
Very preterm	≥90% vs ≤75%-<90%	0.38 (0.23)	.24
Moderately/late preterm	<10% vs ≤75%-<90%	−0.29 (0.09)	.006
Moderately/late preterm	≤10%-<25% vs ≤75%-<90%	−0.14 (0.08)	.24
Moderately/late preterm	≤25%-<50% vs ≤75%-<90%	0.01 (0.07)	.91
Moderately/late preterm	≤50%-<75% vs ≤75%-<90%	0.06 (0.07)	.61
Moderately/late preterm	≥90% vs ≤75%-<90%	−0.02 (0.08)	.91

### Empirically Supported Thresholds for Reduced IQ Performance Based on BWZS and Gestational Age

**Figure 2.**
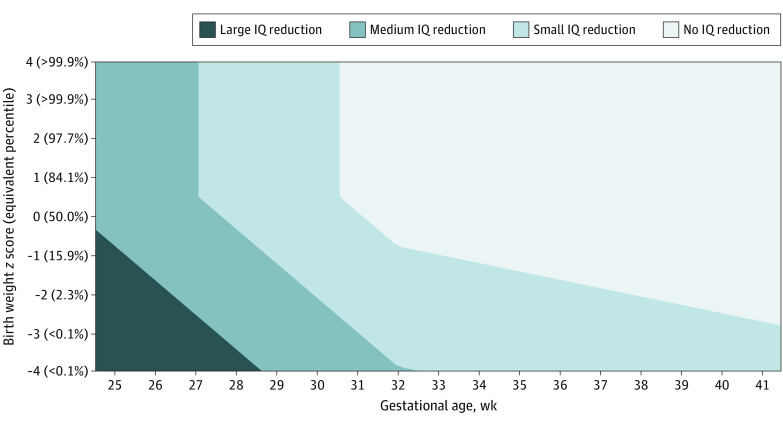
Contour Plot From the Multivariate Adaptive Regression Splines Analysis, Estimated IQ *z *Score by Gestational Age, and Birth Weight *z *Score A large IQ reduction was defined as a *z* score less than −0.8, a medium IQ reduction as a *z* score of −0.8 to less than −0.5, a small IQ reduction as a *z* score of −0.5 to less than −0.2, and no IQ reduction as a *z* score of −0.2 or higher.

From the MARS analysis, estimated IQ scores were calculated based on BWZS and gestational age, determining when IQ scores were associated with small, medium, or large reductions (Figure 2). For an infant born at 37 weeks, a small IQ *z* score reduction (IQ *z* <−0.2) was associated with a BWZS less than −1.9 (BWP <3%). Comparatively, a medium reduction in IQ (IQ *z* score <−0.5) was associated with birth at 33 weeks gestation and with a very small BWZS of –4 (BWP <0.001%). Alternatively, if an infant was born with a BWZS of 0.50 (69th BWP) or above, the estimated IQ Z score was below −0.5 when the infant was born at less than 27 weeks of gestation. Finally, large reductions in IQ were not associated with gestational age until the infant was born at 29 weeks and with a very small BWZS of −4, or if the infant was born at 25 weeks and with a BWZS of −0.85. The results from the MARS analysis indicated that above or below the 10th percentile (*z* = −1.28) would differentiate IQ scores in the normal range from a small IQ reduction, when the infant was born at approximately 34 weeks of gestation.

## Discussion

This study investigated the association of relative birth weight and gestational age with IQ scores in 4 longitudinal cohorts. It was found that IQ scores increased with BWZS until an optimal BWZS of 0.50 (69th BWP), after which there was no association between BWZS and IQ. Independent of BWZS, IQ scores particularly increased from 25 weeks until 32 weeks of gestation with scores continuing to increase, but at a slower rate, up until 41 weeks. It was found that there was no evidence for a turning point where increasing BWZS became negatively associated with IQ performance, nor was there evidence for BWZS interacting with gestational age as a multiplicative risk effect.

The results mostly indicated that “large is good” for IQ. The MARS result indicated that the 69th BWP or above is optimal for IQ, while the multivariable regression using categorical groups similarly indicated that term borns in the 75th to 90th BWP range had the optimal cognitive outcomes. These results are similar to the 66th and 80th percentiles reported previously in Australian and Dutch studies looking at educational outcomes.^8,11^ Furthermore, the present MARS results suggested no reduction in IQ above the 69th BWP, which was similarly found in the multivariable regression where within each gestation group (VP, MLP, and term born), those in the 75th to 90th BWP range did not significantly differ on IQ to those in the 90% or higher. Thus, the present results are more in concordance with the Dutch study where no significant differences above the 80th percentile were found, relative to the Australian study where education scores subsequently declined.^11^ At the lower end of the BWP range, for both term borns and those born MLP, participants in the lowest 10% BWP range had significantly lower IQ than their respective 75th to 90th percentile reference groups, consistent with results indicating SGA is associated with lower cognitive performance.^3^ However, the clinical relevance of this association should be considered. As indicated in Figure 2, term borns do not have an estimated IQ *z* score below −0.2, equivalent to an IQ score of 97, until their BWP is less than the third percentile. This indicates that while dichotomizing at the 10th percentile may find significant IQ differences among term borns, the estimated IQ of SGA term borns will often not be low enough to be of specific concern for clinicians, policymakers, or parents. Regarding VP participants, the 90% and higher BWP group has the highest estimated marginal means within the VP sample, which may suggest that being particularly large for gestational age is a protective factor for IQ of those born VP in a similar way to past findings regarding mortality.^44^ However, no BWP group significantly differed on IQ to those in the 75th to 90th percentile reference group. A lack of significant differences within the VP sample may arise due to lack of statistical power; this should be addressed in future research via the combination of multiple cohorts that specifically recruit those born VP, rather than only using population-based birth cohorts.^45^

Furthermore, every week of gestation was associated with a 1.3 IQ point (0.09 *z* score) increase before reducing in strength to 0.3 IQ points (0.02 *z* score) per week after 32 weeks gestation. This is largely concordant with a previous study in Germany and the UK, where IQ increased by 2.3 points per week until 34 weeks before a complete plateau.^46^ When the overall magnitude of IQ differences between those born VP and term borns is considered, the difference reported herein is smaller than those reported in meta-analyses of cohort studies but larger than those reported from registry studies. Meta-analyses of cohort studies have reported IQ *z* score differences between those born VP and term borns of 0.86^47^ and 0.78,^7^ respectively. In contrast, a Danish registry study using a sibling-matched approach indicated a much smaller difference at just 0.25 for adolescent boys born between 28 and 31 weeks gestation.^48^ By uniquely testing for nonlinear associations, the current study indicates that at 32 weeks of gestation the estimated IQ *z* score was −0.13, which then changed dramatically due to the hinge point to approximately −0.50 at 28 weeks. Additional methodological differences should be considered, including the use of multiple different cognitive tests rather than using a fully validated IQ test or that proxy scores were not calculated for participants unable to take part due to impairments. Because participants with lower gestational ages were more likely to have impairments that prevented them from completing the test, the present estimate of the IQ difference between those born VP and term borns may be an underestimation of the true population difference.^48,49^

### Limitations

Rather than using hospital records, birth weight and gestational age were parent reported. However, in the year after birth, more than 90% of parents can accurately recall their child’s birth weight within 100 g^28^ and a week of gestation.^29^ This also meant that we could not investigate potentially important neonatal morbidities for IQ, such as intraventricular hemorrhage or bronchopulmonary dysplasia,^7,47^ or differentiate constitutionally small infants from those with intrauterine growth restriction, which may be stronger evidence of pathology.^50,51^ However, the relative effect sizes of neonatal morbidities have been found to be smaller than socioenvironmental factors.^7^ While socioenvironmental factors were thoroughly controlled for, there may be further potentially important confounding factors such as familial dietary patterns that should be considered.^52^ Overall, gestational age and relative birth weight should potentially be thought of as early and easily measurable indicators that an infant is at risk for lowered childhood IQ and potentially beyond, given reports of stable associations between preterm birth and SGA with IQ differences into adulthood.^4,53^

## Conclusions

In this cohort study of 4 large, longitudinal cohorts, increasing BWP was associated with increased IQ until a plateau at the 69th BWP, and this association was independent of gestational age’s association with IQ. When considering both factors additively, being born at 34 weeks gestation and below the 10th BWP was associated with a small IQ reduction. For term borns, a lower BWP cutoff at or below the third BWP was associated with a similarly small IQ reduction.

## Notes

Supplement 1. **eTable 1. **Attrition Data in the Four Cohorts (Only Target sample) **eTable 2.** Logistic regressions testing for Systematic drop out within each cohort (Target Sample) **eFigure 1.** Growing up in Ireland flow chart **eFigure 2.** Longitudinal Study of Australian Children flow chart **eFigure 3. **Millennium Cohort Study flow chart **eFigure 4. **National Longitudinal Survey of Youth 1979 Child and Adolescence flow chart **eTable 3. **Comparison of the multivariable regression predicting IQ Z scores using categorical groups for BWP and gestational age (left) relative to the MARS analysis using continuous gestational age and BWZS (right)

Supplement 2.
Data Sharing Statement
