# Supplementary material for: Association of Birth Weight Centiles and Gestational Age With Cognitive Performance at Age 5 Years
Source: JAMA Netw Open. 2023 Aug 31;6(8):e2331815. doi: 10.1001/jamanetworkopen.2023.31815 (PMC10472194; doi:10.1001/jamanetworkopen.2023.31815)
Supplement: Supplement 1. — eTable 1. Attrition Data in the Four Cohorts (Only Target sample) eTable 2. Logistic regressions testing for Systematic drop out within each cohort (Target Sample) eFigure 1. Growing up in Ireland flow chart eFigure 2. Longitudinal Study of Australian Children flow chart eFigure 3. Millennium Cohort Study flow chart eFigure 4. National Longitudinal Survey of Youth 1979 Child and Adolescence flow chart eTable 3. Comparison of the multivariable regression predicting IQ Z scores using categorical groups for BWP and gestational age (left) relative to the MARS analysis using continuous gestational age and BWZS (right) [file jamanetwopen-e2331815-s001.pdf]

Supplemental Online Content

Eves R, Wolke D, Spiegler J, Lemola S. Association of birth weight centiles and gestational age with cognitive performance at age 5 years. *JAMA Netw Open*. 2023;6(8):e2331815. doi:10.1001/jamanetworkopen.2023.31815

eTable 1. Attrition Data in the Four Cohorts (Only Target sample)

eTable 2. Logistic regressions testing for Systematic drop out within each cohort (Target Sample)

eFigure 1. Growing up in Ireland flow chart

eFigure 2. Longitudinal Study of Australian Children flow chart

eFigure 3. Millennium Cohort Study flow chart

eFigure 4. National Longitudinal Survey of Youth 1979 Child and Adolescence flow chart

eTable 3. Comparison of the multivariable regression predicting IQ Z scores using categorical groups for BWP and gestational age (left) relative to the MARS analysis using continuous gestational age and BWZS (right)

This supplemental material has been provided by the authors to give readers additional information about their work.

© 2023 Eves R et al. *JAMA Netw Open.*

eTable 1. Attrition Data in the Four Cohorts (Only Target sample)

	GUI (Ireland)		LSAC (Australia)		MCS (UK)		NLSY79 (USA)	
	**Drop Out (N=1814)**	**Participating Age 5 (N=7290)**	**Drop Out (N=1037)**	**Participating Age 5 (N=3738)**	**Drop Out (N=3739)**	**Participating Age 5 (N=14072)**	**Drop Out (N=2243)**	**Participating Age 5 (N=5543)**
**Gestational Age (groups)**								
Very Preterm	0 (0%)	0 (0%)	12 (1.2%)	26 (0.7%)	56 (1.5%)	149 (1.1%)	49 (2.2%)	81 (1.5%)
Moderately/ Late Preterm	84 (4.6%)	228 (3.1%)	71 (6.8%)	203 (5.4%)	271 (7.2%)	984 (7.0%)	255 (11.4%)	617 (11.1%)
Term Born	1730 (95.4%)	7062 (96.9%)	954 (92.0%)	3509 (93.9%)	3412 (91.3%)	12939 (91.9%)	1939 (86.4%)	4845 (87.4%)
**Gestational Age (weeks)**								
Mean (SD)	39.4 (1.42)	39.5 (1.34)	38.9 (2.02)	39.1 (1.78)	39.2 (2.08)	39.3 (1.97)	38.4 (2.18)	38.5 (1.93)
**Child Sex**								
Male	967 (53.3%)	3679 (50.5%)	521 (50.2%)	1916 (51.3%)	1956 (52.3%)	7164 (50.9%)	1160 (51.7%)	2810 (50.7%)
Female	847 (46.7%)	3611 (49.5%)	516 (49.8%)	1822 (48.7%)	1783 (47.7%)	6908 (49.1%)	1083 (48.3%)	2733 (49.3%)
**Fenton Birthweight Z score**								
Mean (SD)	-0.0168 (0.937)	0.0667 (0.892)	-0.124 (0.977)	0.0000673 (0.930)	-0.225 (0.983)	-0.184 (0.984)	-0.0232 (1.04)	0.0474 (1.06)
**Household Income**								

© 2023 Eves R et al. *JAMA Netw Open.*

	GUI (Ireland)		LSAC (Australia)		MCS (UK)		NLSY79 (USA)	
	**Drop Out (N=1814)**	**Participating Age 5 (N=7290)**	**Drop Out (N=1037)**	**Participating Age 5 (N=3738)**	**Drop Out (N=3739)**	**Participating Age 5 (N=14072)**	**Drop Out (N=2243)**	**Participating Age 5 (N=5543)**
Low Income	801 (44.2%)	2306 (31.6%)	457 (44.1%)	941 (25.2%)	1899 (50.8%)	5079 (36.1%)	497 (22.2%)	1178 (21.3%)
Medium Income	524 (28.9%)	2644 (36.3%)	325 (31.3%)	1544 (41.3%)	1114 (29.8%)	4925 (35.0%)	1094 (48.8%)	2674 (48.2%)
High Income	309 (17.0%)	1828 (25.1%)	186 (17.9%)	1075 (28.8%)	701 (18.7%)	4037 (28.7%)	652 (29.1%)	1691 (30.5%)
Missing	180 (9.9%)	512 (7.0%)	69 (6.7%)	178 (4.8%)	25 (0.7%)	31 (0.2%)	0 (0%)	0 (0%)
**Maternal Education**								
Non-University Educated	853 (47.0%)	3883 (53.3%)	813 (78.4%)	2387 (63.9%)	3337 (89.2%)	11620 (82.6%)	1498 (66.8%)	3660 (66.0%)
University Educated	401 (22.1%)	1980 (27.2%)	224 (21.6%)	1351 (36.1%)	382 (10.2%)	2424 (17.2%)	745 (33.2%)	1883 (34.0%)
Missing	560 (30.9%)	1427 (19.6%)	0 (0%)	0 (0%)	20 (0.5%)	28 (0.2%)	0 (0%)	0 (0%)

© 2023 Eves R et al. *JAMA Netw Open.*

eTable 2. Logistic regressions testing for Systematic drop out within each cohort (Target Sample)

	**GUI Drop Out**			**LSAC Drop Out**			**MCS Drop Out**			**NLSY79 Drop Out**		
Predictors	**Odds Ratios**	**CI**	**p**	**Odds Ratios**	**CI**	**p**	**Odds Ratios**	**CI**	**p**	**Odds Ratios**	**CI**	**p**
(Intercept)	3.68	0.80 – 16.62	0.092	2.78	0.68 – 11.00	0.149	0.88	0.44 – 1.73	0.718	1.16	0.45 – 2.95	0.758
Fenton Birthweight Z	0.88	0.83 – 0.93	**<0.001**	0.86	0.80 – 0.93	**<0.001**	0.95	0.92 – 0.99	**0.010**	0.93	0.89 – 0.97	**0.002**
Gestational age (weeks)	0.93	0.90 – 0.97	**<0.001**	0.94	0.91 – 0.98	**0.001**	0.97	0.95 – 0.99	**<0.001**	0.97	0.95 – 1.00	**0.028**
Observations	9104			4775			17811			7786		
R^2 Tjur	0.003			0.005			0.001			0.002		

© 2023 Eves R et al. *JAMA Netw Open.*

eFigure 1. Growing up in Ireland flow chart

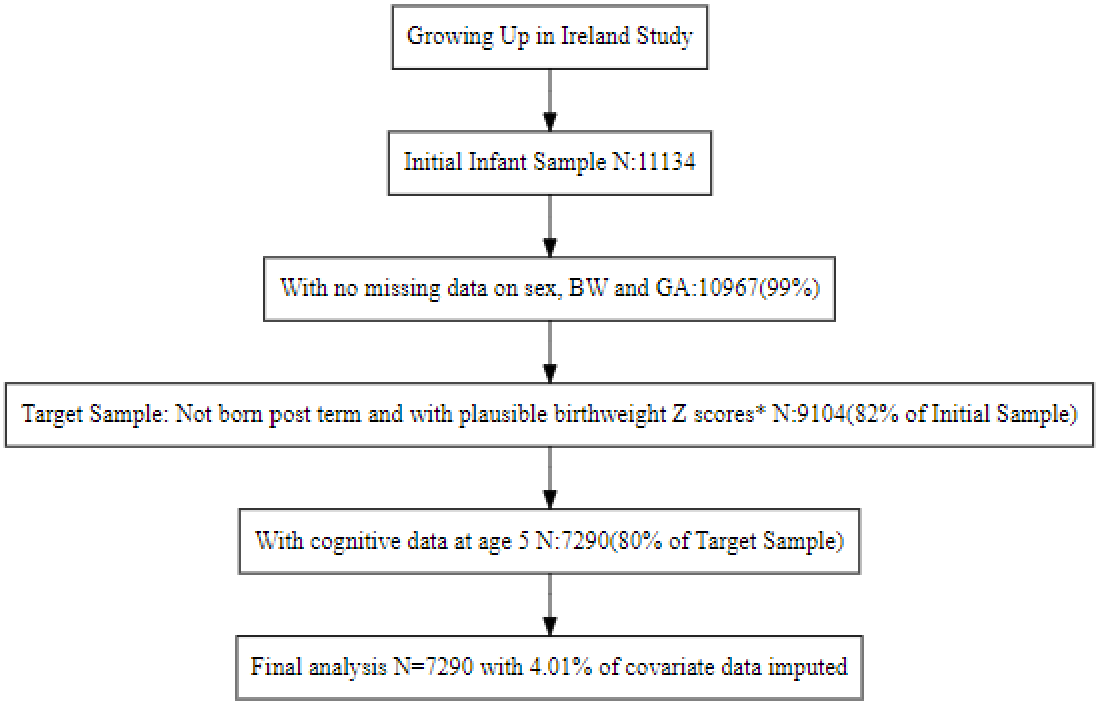

*Target Sample in the GUI also does not include infants with a weight below 2500g.

© 2023 Eves R et al. *JAMA Netw Open.*

eFigure 2. Longitudinal Study of Australian Children flow chart

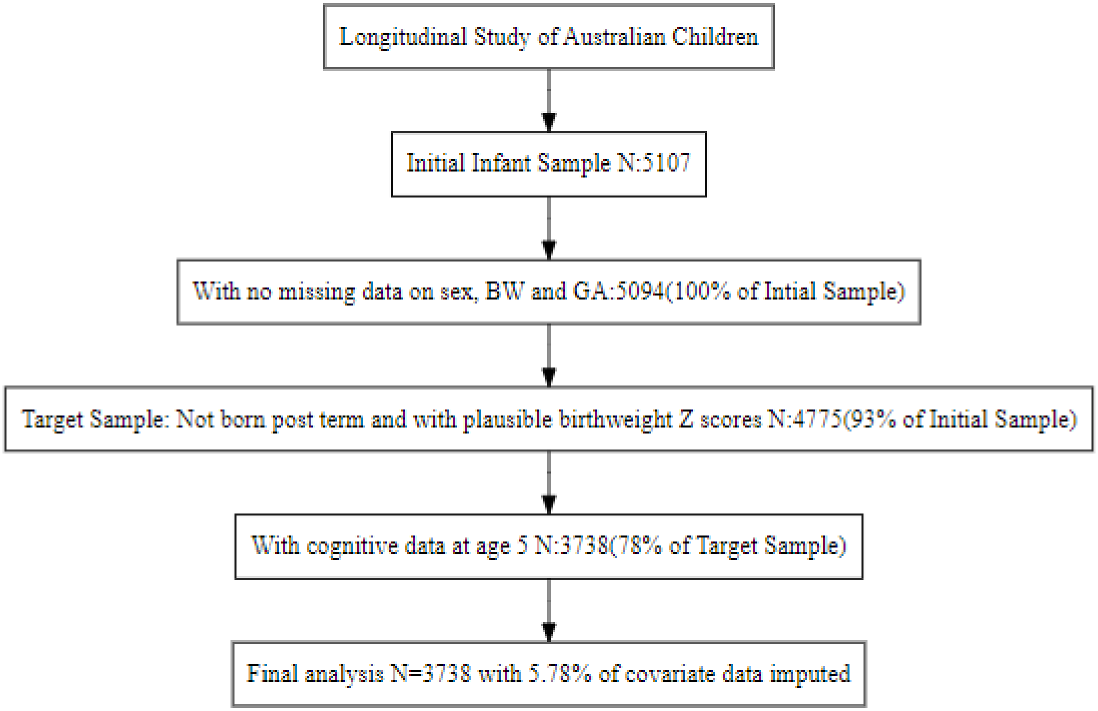

© 2023 Eves R et al. *JAMA Netw Open.*

eFigure 3. Millennium Cohort Study flow chart

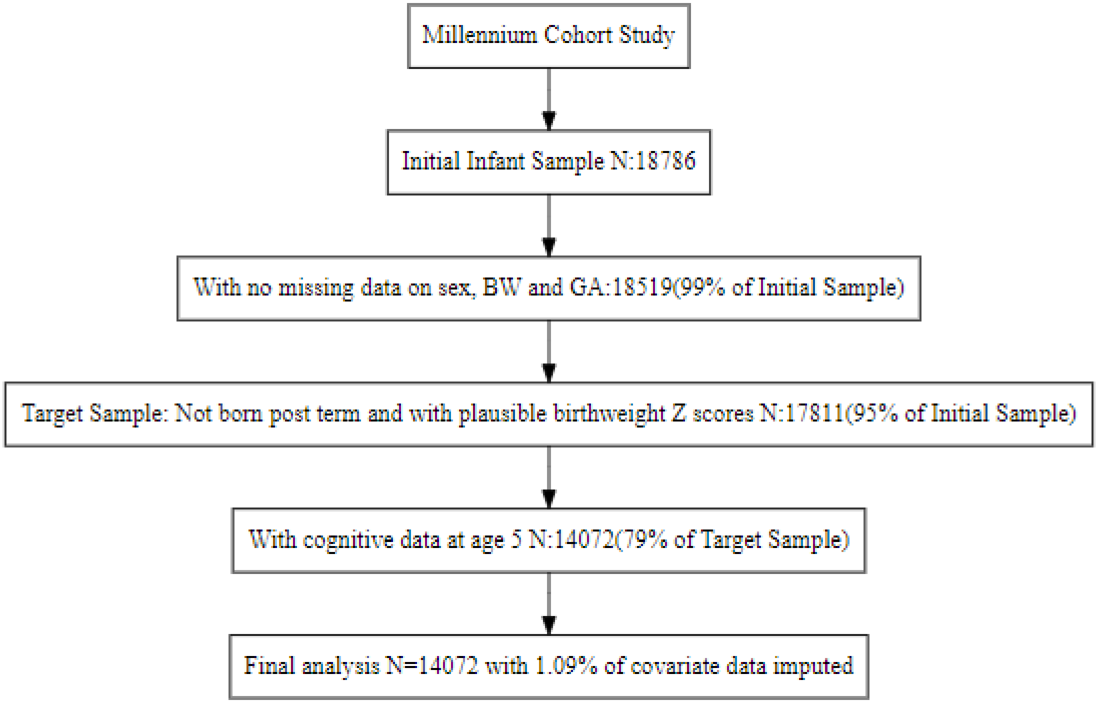

© 2023 Eves R et al. *JAMA Netw Open.*

eFigure 4. National Longitudinal Survey of Youth 1979 Child and Adolescence flow chart

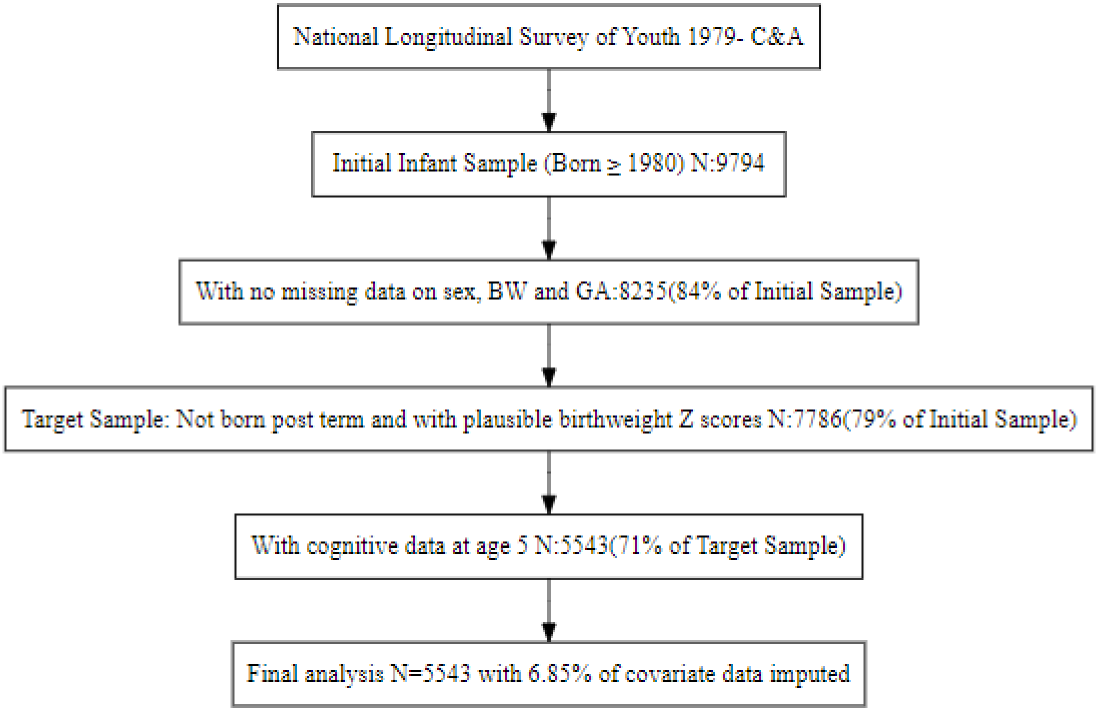

© 2023 Eves R et al. *JAMA Netw Open.*

eTable 3. Comparison of the multivariable regression predicting IQ Z scores using categorical groups for BWP and gestational age (left) relative to the MARS analysis using continuous gestational age and BWZS (right)

		Multivariable Regression			MARS Analysis	
**Feature**	**beta estimate**	**CI**	**P value**	**beta estimate**	**CI**	**P value**
Intercept	-0.75	-1.02 – -0.48	≤0.001	-0.14	-0.23 – -0.05	**0.003**
Very Preterm (ref:Term)	-0.50	-0.80 – -0.20	0.01	-	-	-
MLP(ref:Term)	-0.09	-0.20 – 0.01	0.09	-	-	-
BWP <10%(ref:75-90%)	-0.21	-0.26 – -0.17	≤0.001	-	-	-
BWP 10-25%(ref:75-90%)	-0.12	-0.16 – -0.08	≤0.001	-	-	-
BWP 25-50%(ref:75-90%)	-0.06	-0.10 – -0.03	≤0.001	-	-	-
BWP 50-75%(ref:75-90%)	-0.02	-0.05 – 0.02	0.38	-	-	-
BWP >90%(ref:75-90%)	-0.02	-0.07 – 0.03	0.37	-	-	-
Gestational Age up to 32 weeks	-	-	-	0.09	0.05 – 0.12	**<0.001**
Gestational Age after 32 weeks	-	-	-	0.02	0.01 – 0.03	**<0.001**
BWZS until Z =0.50 (69th percentile)	-	-	-	0.10	0.08 – 0.11	**<0.001**
Maternal Height (cm)	0.00	0.00 – 0.01	≤0.001	Non Meaningful	Non Meaningful	Non Meaningful

© 2023 Eves R et al. *JAMA Netw Open.*

	Multivariable Regression			MARS Analysis		
**Feature**	**beta estimate**	**CI**	**P value**	**beta estimate**	**CI**	**P value**
Maternal Weight (kg)	-0.00	-0.00 – -0.00	≤0.001	-0.00	-0.00 – -0.00	**<0.001**
Family Income- Medium (ref:Low)	0.22	0.20 – 0.25	≤0.001	0.23	0.20 – 0.25	**<0.001**
Family Income- High (ref:low)	0.37	0.34 – 0.40	≤0.001	0.39	0.36 – 0.42	**<0.001**
Maternal Age (years)	0.01	0.01 – 0.01	≤0.001	0.01	0.01 – 0.01	**<0.001**
University Educated Mother (ref:non-university)	0.25	0.22 – 0.27	≤0.001	0.24	0.21 – 0.27	**<0.001**
Maternal Parity - multiparous(ref:primiparous)	-0.23	-0.25 – -0.21	≤0.001	-0.23	-0.25 – -0.21	**<0.001**
Primary Language non-english (ref: English)	-0.57	-0.60 – -0.53	≤0.001	-0.57	-0.61 – -0.54	**<0.001**
Mother Not Married (ref: married)	-0.11	-0.14 – -0.08	≤0.001	-0.11	-0.13 – -0.08	**<0.001**
Child Sex- Male (ref:Female)	-0.13	-0.15 – -0.11	≤0.001	-0.13	-0.15 – -0.11	**<0.001**
Cohort LSAC (ref: GUI)	-0.03	-0.07 – 0.01	0.11	Non Meaningful	Non Meaningful	Non Meaningful
Cohort MCS (ref: GUI)	0.08	0.05 – 0.10	≤0.001	Non Meaningful	Non Meaningful	Non Meaningful
Cohort NLSY79 (ref: GUI)	0.08	0.05 – 0.12	≤0.001	Non Meaningful	Non Meaningful	Non Meaningful
Very Preterm*BWP<10%	-0.05	-0.49 – 0.40	0.84	-	-	-
MLP*BWP<10%	-0.08	-0.26 – 0.11	0.41	-	-	-

© 2023 Eves R et al. *JAMA Netw Open.*

		Multivariable Regression			MARS Analysis	
**Feature**	**beta estimate**	**CI**	**P value**	**beta estimate**	**CI**	**P value**
Very Preterm*BWP 10-25%	0.09	-0.35 – 0.52	0.70	-	-	-
MLP*BWP 10-25%	-0.02	-0.18 – 0.15	0.84	-	-	-
Very Preterm*BWP 25-50%	0.14	-0.24 – 0.52	0.47	-	-	-
MLP Preterm*BWP 25-50%	0.07	-0.07 – 0.21	0.31	-	-	-
Very Preterm*BWP 50-75%	0.07	-0.32 – 0.45	0.73	-	-	-
MLP*BWP 50-75%	0.07	-0.06 – 0.21	0.30	-	-	-
Very Preterm*BWP >90%	0.40	-0.05 – 0.84	0.08	-	-	-
MLP*BWP >90%	0.00	-0.15 – 0.16	0.95	-	-	-

Multi adaptive regression spline (MARS), Birthweight Percentile (BWP), Birthweight Z Score (BWZS), Moderately/Late Preterm (MLP), Growing Up in Ireland Study (GUI), Millennium Cohort Study (MCS), Longitudinal Study of Australian Children (LSAC), National Longitudinal Survey of Youth 1979 – Child Sample (NLSY79).

Note: Producing confidence intervals and P-values for MARS analyses is generally discouraged. As the optimal model is developed using cross-validation, which optimises the variance explained on the test datasets, it is a "self-fulfilling prophecy" that these variables will be highly significant when the features selected are tested as part of a multivariable regression. See here for further details: http://www.milbo.org/doc/earth-notes.pdf [1]
