# Supplementary material for: Association of Birth Weight Centiles and Gestational Age With Cognitive Performance at Age 5 Years
Source: JAMA Netw Open. 2023 Aug 31;6(8):e2331815. doi: 10.1001/jamanetworkopen.2023.31815 (PMC10472194; doi:10.1001/jamanetworkopen.2023.31815)
Supplement: Supplement 2. — Data Sharing Statement [file jamanetwopen-e2331815-s002.pdf]

Data Sharing Statement

Eves. Association of Birth Weight Centiles and Gestational Age With Cognitive Performance at Age 5 Years. *JAMA Netw Open*. Published August 31, 2023.
doi:10.1001/jamanetworkopen.2023.31815

Data
Data available: No

Additional Information
Explanation for why data not available: The individual-level data is publicly available under license but must be requested from each cohort's specific website. All data are open access and can be accessed at the following websites: MCS- https://cls.ucl.ac.uk/cls-studies/millennium-cohort-study/. GUI: https://www.growingup.gov.ie/. LSAC: https://growingupinaustralia.gov.au/. NLSY79: https://www.nlsinfo.org/content/cohorts/nlsy79-children. The code used specifically for this analysis is available on the Open Science Framework: https://osf.io/ybcnm/
